# Chronological Changes of Lower Urinary Tract Symptoms in Elderly Patients with Prostate Cancer after Low-Dose-Rate Prostate Brachytherapy

**DOI:** 10.3390/life13071507

**Published:** 2023-07-04

**Authors:** Kunihiro Tsuchiya, Makoto Kawase, Keita Nakane, Masahiro Nakano, Koji Iinuma, Daiki Kato, Manabu Takai, Yuki Tobisawa, Takayuki Mori, Hirota Takano, Tomoyasu Kumano, Masayuki Matsuo, Takayasu Ito, Takuya Koie

**Affiliations:** 1 Department of Urology, General Home Care Clinic, Gifu 5016014, Japan; ktsuchiya@iroha-care.com; 2 Department of Urology, Gifu University Graduate School of Medicine, Gifu 5011194, Japan; nakane.keita.k2@f.gifu-u.ac.jp (K.N.); iinuma.koji.s0@f.gifu-u.ac.jp (K.I.); kato.daiki.m2@f.gifu-u.ac.jp (D.K.); takai.manabu.a5@f.gifu-u.ac.jp (M.T.); tobisawa.yuki.a7@f.gifu-u.ac.jp (Y.T.); koie.takuya.h2@f.gifu-u.ac.jp (T.K.); 3 Department of Urology, Gifu Prefectural General Medical Center, Gifu 5008717, Japan; nakano-m@juno.ocn.ne.jp; 4 Department of Radiology, Gifu University Graduate School of Medicine, Gifu 5011194, Japan; mori.takayuki.b4@f.gifu-u.ac.jp (T.M.); takano.hirota.w2@f.gifu-u.ac.jp (H.T.); kumano.tomoyasu.k0@f.gifu-u.ac.jp (T.K.); matsuo.masayuki.e0@f.gifu-u.ac.jp (M.M.); 5 Center for Clinical Training and Career Development, Gifu University Graduate School of Medicine, Gifu 5011194, Japan; ito.takayasu.v9@f.gifu-u.ac.jp

**Keywords:** prostate cancer, low-dose-rate brachytherapy, elderly patients, lower urinary tract symptoms, overactive bladder symptom score

## Abstract

Background: To compare chronological changes in lower urinary tract symptoms (LUTS) after low-dose-rate prostate extended-release therapy (LDR-BT) using the overactive bladder symptom score (OABSS) in patients aged ≥ 75 years (elderly group) versus those aged < 75 years (control group). Materials and Methods: Patients with prostate cancer who underwent LDR-BT at Gifu University Hospital were included in this study. The International Prostate Symptom Score (IPSS), OABSS, and quality of life-based on urinary symptoms (IPSS-QOL) were evaluated before and after LDR-BT. We compared chronological changes in IPSS, OABSS, and IPSS-QOL in the elderly group with those in the control group and assessed the association between the resolution of OABSS and clinicopathological covariates. Results: A total of 484 patients were enrolled in this study. In the elderly group, the total IPSS, OABSS, and frequency scores increased at 1 month postoperatively, whereas the control group showed an increase at 3 months postoperatively. Multivariate analysis identified changes from baseline to the maximum OABSS and pre-treatment OABSS as significant predictors of delayed resolution of OABSS after LDR-BT. Conclusions: Changes in pre-treatment OABSS and pre- and post-LDR-BT OABSS values were independent predictors of delayed resolution of OABSS; however, no correlation was found with age.

## 1. Introduction

Prostate cancer (PCa) is the most common cancer in men, with an estimated 1,414,259 men diagnosed and 375,304 deaths from PCa in 2020, making it the fifth leading cause of cancer-related deaths [1]. Among them, 708,506 men were diagnosed with PCa, and 289,252 died from PCa in 2020 when limited to those aged ≥ 70 years, making it the second leading cause of cancer-related deaths among men [1]. The National Comprehensive Cancer Network (NCCN) guidelines in the United States recommend definitive therapy for unfavorable intermediate-risk PCa with a life expectancy of >10 years and for high- and very-high-risk PCa with a life expectancy of >5 years [2]. Owing to the rapid increase in the elderly population and extended life expectancy in Japan, definitive therapy should be considered for PCa, even in elderly patients [3].

Low-dose-rate brachytherapy with iodine-125 (LDR-BT) is a definitive treatment modality for localized and selected locally advanced PCa, with excellent oncologic outcomes comparable to those of radical prostatectomy (RP) and external-beam radiation therapy (EBRT) [4,5]. Furthermore, the combination of LDR-BT and EBRT has been recognized as a potential treatment for improving oncologic outcomes in patients with PCa because it allows for an increased dose to the prostate [5,6]. In elderly patients, LDR-BT has shown relatively good control with respect to biochemical recurrence-free survival (BRFS) [7]. However, the incidence of acute and late treatment-related complications, particularly genitourinary (GU) toxicity, is relatively high [8,9]. A total of 67.2% of patients treated with LDR-BT alone and 50.3% of those treated with a combination of LDR-BT and EBRT were reported to have GU toxicity; 53% and 42%, respectively, were reported to have frequent urination or urinary urgency [8,9]. The International Prostate Symptom Score (IPSS) is the most widely used tool to score and assess urinary storage and voiding symptoms, respectively [10]. The overactive bladder symptom score (OABSS) is a questionnaire that assesses the severity of overactive bladder (OAB) using four questions (question 1: storage symptoms during daytime voiding; question 2: storage symptoms during nighttime voiding; question 3: urgency and question 4: urge incontinence) [11]. It is known that IPSS, OABSS, and quality of life by urinary symptom (IPSS-QOL) scores worsen immediately after LDR-BT compared with preoperative scores, although these symptoms return to baseline approximately 18–36 months after surgery in patients who underwent LDR-BT [12]. However, voiding symptoms may take longer to return to baseline or may not reach baseline compared to voiding symptoms, especially in elderly patients [13,14].

Thus, we aimed to evaluate the chronological changes in lower urinary tract symptoms (LUTS) over time after LDR-BT for patients ≥ 75 years of age (elderly group), especially with regard to the change in total OABSS and each OABSS item, compared to those <75 years of age (control group).

## 2. Materials and Methods

### 2.1. Patient Selection

This approval for this study was obtained from the Institutional Review Board of Gifu University (approval number: 2020-210). As this was a retrospective study, the requirement for informed consent was waived. In accordance with the provisions of the Japanese Ethics Committee and Ethics Guidelines, written consent was not required because the results of the retrospective and observational studies using existing materials and other data have already been made publicly available. Details of this study, which are available only in Japanese, can be found at https://www.med.gifu-u.ac.jp/visitors/disclosure/docs/2020-210.pdf (accessed on 29 May 2023).

We conducted a retrospective cohort study of consecutive patients with PCa at clinical stages T1c/T2/T3a who were diagnosed according to the 2018 American Joint Committee on Cancer staging manual [15] and underwent LDR-BT at Gifu University Hospital between August 2000 and December 2015. All enrolled patients were stratified into very-low-, low-, favorable intermediate-, unfavorable intermediate-, high-, and very-high-risk groups based on the risk classification of the NCCN guidelines (version 4, 2022) [2]. Until 2005, LDR-BT was selected as a treatment for low- and intermediate-risk PCa in almost all patients, while EBRT was performed for high-risk PCa; since 2006, the combination of LDR-BT and EBRT for high-risk PCa has increased. On the other hand, surgery was rarely performed during the observation period.

### 2.2. Treatment

Patients were placed with loose 125I radioactive seeds (Oncoseed, Nihon Medi-physics, Tokyo, Japan) using the Mick applicator (Mick Radio-Nuclear Instruments, Bronx, NY, USA) or inserted with the ProLink^®^ delivery system (C. R. Bard, Inc., Murray Hill, NJ, USA) using a real-time rectal ultrasound-guided transperineal technique [16]. The prescribed minimal peripheral dose to the prostate was 145 Gy for patients who received LDR-BT alone and 104 Gy for patients who received a combination of LDR-BT and EBRT. When EBRT was performed, the prostate and seminal vesicles were irradiated in 2 Gy fractions for a total of 40 Gy within 1 month after LDR-BT. Neoadjuvant androgen deprivation therapy (NADT) was administered for at least 3 months to very-low- and low-risk patients with PCa who had a pre-treatment prostate volume (PV) of >50 mL in an attempt to reduce PV. Postoperative ADT was not administered to these patients. All patients with favorable and unfavorable intermediate-risk PCa received androgen deprivation therapy (ADT) in combination with LDR-BT and/or EBRT for 9 months. All patients with high- and very-high-risk PCa were also treated with LDR-BT and EBRT in addition to 24 months of ADT. To reduce the risks of urinary retention and LUTS, α-1 blockers were routinely administered after LDR-BT. In all cases, pre-planning was carried out before seed implantation using a modified peripheral loading technique [17].

### 2.3. Post-Dosimetric Evaluation

Treatment planning and post-implant dosimetry were performed using the latest American Association of Physicians in Medicine Task Group 43 formalism and Variseed version 7.1 (Varian Medical Systems, Palo Alto, CA, USA). A post-implant dosimetric study using computed tomography (CT) and magnetic resonance imaging (MRI) was performed 1 month after LDR-BT. CT was performed using a CT scanner with a 16 or 64 detector array (LightSpeed Ultra 16/Discovery CT 750 HD; GE Healthcare, Milwaukee, WI, USA), and MRI under respiratory depression was performed using a five-channel SENSE cardiac coil with a slice thickness of 3 mm and no cross-gap (Intera Achieva 1.5 T/Intra Achieva Nova Dual 1.5 T Pulsar; Philips Medical Systems, Eindhoven, The Netherlands). The dosimetric parameters analyzed in this study were the biological effective dose (BED), minimum dose received by 90% of the target volume (D90), percentage of target volume receiving at least 100% of the prescribed dose (V100), minimum dose received by 30% of the urethral volume (UD30), and rectal volume receiving 100% of the prescribed dose (RV100).

### 2.4. Follow-Up Schedule

After LDR-BT, all patients had serum PSA and testosterone levels measured at 3–6 month intervals for the first 5 years and then at 6–12 month intervals thereafter for biological recurrence (BCR). BCR after LDR-BT was defined as an increase in PSA > 2 ng/mL from the nadir value based on the Radiation Therapy Oncology Group-Phoenix definition [18]. The so-called PSA bounce, a temporary increase in PSA, was not diagnosed as a BCR. The IPSS, OABSS, and IPSS-QOL were measured before LDR-BT and at 1, 3, 6, 9, 12, 18, 24, 36, 48, and 60 months after LDR-BT.

### 2.5. Endpoints and Statistical Analysis

The primary endpoint of the study was the time to resolution of IPSS, OABSS, and IPSS-QOL in the elderly group compared to the control group. The secondary endpoint was the association between the resolution of OABSS and clinicopathological covariates. Resolution was defined as the return of IPSS and OABSS to baseline scores after LDR-BT. Patient characteristics were described as the median and interquartile range (IQR) for continuous variables and counts and proportions for categorical variables. Fisher’s exact test was used to compare categorical variables, and the Wilcoxon signed-rank test was used to compare continuous variables. Chronological changes in IPSS, OABSS, and IPSS-QOL are represented using line graphs with median and interquartile range (IQR) values. For each factor of the OABSS, the mean ± standard deviation (SD) was used to evaluate chronological changes during the follow-up period. The resolution of the OABSS was analyzed using the Kaplan–Meier method. The resolution of OABSS in both groups was analyzed using the log-rank test. Multivariate analysis was performed using a Cox proportional hazards model. All *p*-values were two-tailed, and a *p*-value < 0.05 was considered statistically significant. JMP Pro 16 (SAS Institute Inc., Cary, NC, USA) was used for the data analysis.

## 3. Results

### 3.1. Patient Characteristics

Table 1 shows the patient characteristics and dosimetric data for LDR-BT. A total of 484 patients met the inclusion criteria. Patients with lymph node involvement, distant metastases, prior transurethral resection of the prostate, or uroflowmetry (UFM) evaluation with a maximal flow rate (Qmax) of <10 mL/s were excluded from the study. The median follow-up period for the entire cohort was 73.0 months (IQR: 41.7–111.2 months), and the median age of all patients was 66 years (IQR: 62–71 years). The median initial PSA was 6.5 ng/mL (IQR: 5.1–9.1 ng/mL) and the Gleason grade group was 2 (IQR: 1–2). A total of 375 patients received NADT before LDR-BT, and 199 patients were treated with a combination of LDR-BT and EBRT. Compared with the control group, the elderly group had statistically smaller PVs, higher PSA values, and a higher ratio of unfavorable intermediate- and high-risk PCa based on the NCCN classification. Regarding the prescribed doses, there were no significant differences in BED, D90, V100, and RV100 between the two groups; however, UD30 was significantly lower in the elderly group than in the control group.

### 3.2. Oncological Outcomes

**Figure 1:**
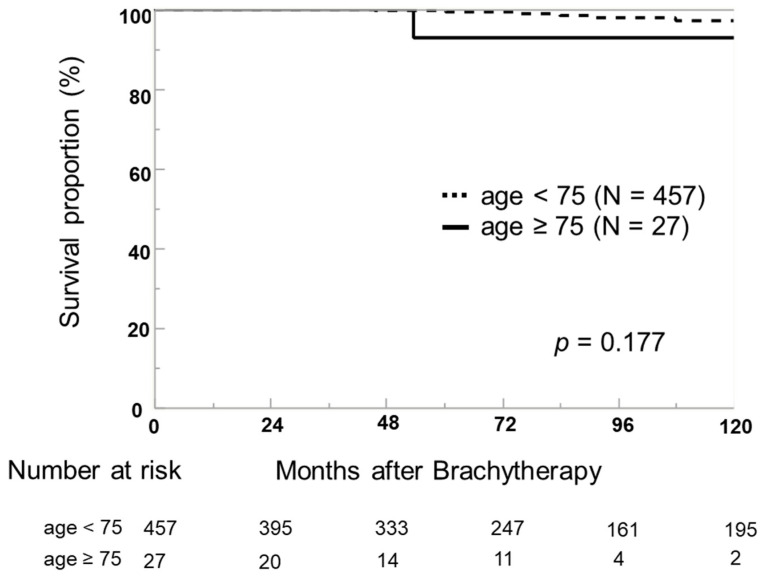
Kaplan–Meier estimates of biochemical recurrence-free survival (BRFS) according to patients’ age at the time of low dose rate brachytherapy with iodine-125. The 5- and 10-year BRFS rates in patients aged <75 years were 99.7% and 97.2%, respectively. The 5- and 10-year survival rates in patients aged ≥75 years were 92.9% and 92.9%, respectively. There was no statistical significance between the two groups.

There were no deaths due to PCa among the enrolled patients during the follow-up period; however, BCR was observed in ten patients (2.1%). Figure 1 shows the BRFS. The 5- and 10-year BRFS rates in the elderly group were 92.9% and 92.9%, respectively. The 5- and 10-year BRFS rates in the control group were 99.7% and 97.2%, respectively. There was no significant difference in BRFS between the two groups (*p* = 0.177).

### 3.3. Chronological Changes in IPSS, OABSS, and IPSS-QOL

**Figure 2:**
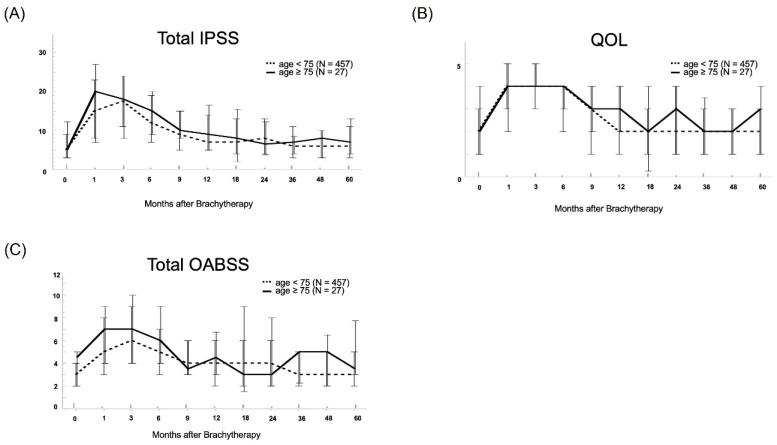
Linear mixed-effects model showed chronological changes in the following lower urinary tract symptom scores: (**A**) IPSS total, (**B**) IPSS-QOL, and (**C**) OABSS total. In patients <75 years, each score increased at 3 months after low dose rate brachytherapy with iodine-125 (LDR-BT), whereas in those ≥75 years, the total IPSS and OABSS scores increased at 1 month after LDR-BT. Additionally, each score required a period of 18–36 months to improve to baseline values. Although there were no significant differences in IPSS, OABSS, or IPSS-QOL over time during the follow-up period, only the pre-treatment OABSS score was significantly different between the two groups (*p* = 0.014).

Figure 2 shows the linear mixed-effects models for chronological changes in IPSS, OABSS, and IPSS-QOL. In the control group, each score increased 3 months after LDR-BT, whereas in the elderly group, the sum of the IPSS and OABSS scores increased 1 month after LDR-BT.

**Figure 3:**
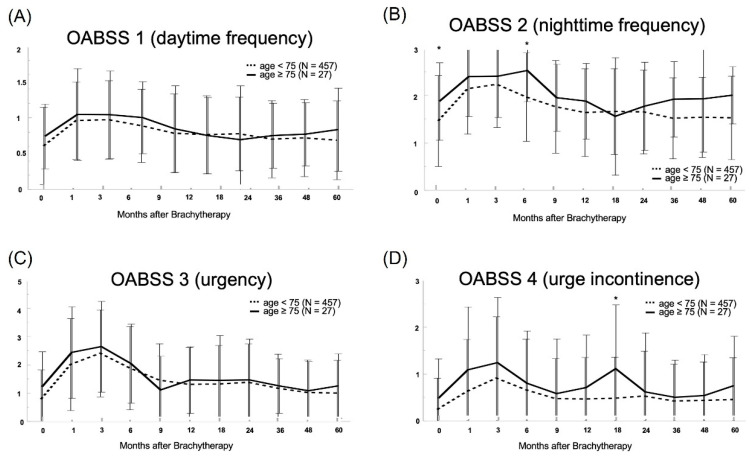
Chronological change in each of the following OABSS scores from a linear mixed-effects model: (**A**) daytime frequency, (**B**) nighttime frequency, (**C**) urgency, and (**D**) urgency incontinence. In both groups, urgency and urge urinary incontinence scores increased at 3 months after low dose rate brachytherapy with iodine-125 (LDR-BT), whereas daytime frequency scores increased at 1 month after LDR-BT. Significant differences were found between the two groups in nighttime frequency before and 6 months after LDR-BT and in urge incontinence at 18 months (*p* = 0.046, *p* = 0.004, *p* = 0.041, respectively) * *p* < 0.05.

Figure 3 shows the chronological changes in the four sub-scores of the OABSS. The OABSS showed higher nighttime frequency and urge urinary values in the elderly group than in the control group.

The relationship between the postoperative follow-up period and the cumulative resolution rate of the OABSS was examined in both groups. The resolution rates of the OABSS at 1, 3, and 5 years were 47.2%, 67.4%, and 74.6% in the control group, and 53.9%, 90.8%, and 90.8% in the elderly group. There was no statistically significant difference between the two groups in terms of OABSS resolution rate (*p* = 0.285).

Multivariate analysis identified preoperative OABSS and the rate of change in OABSS from baseline as significant predictors of OABSS resolution after LDR-BT (Table 2). However, no association was observed between age and OABSS resolution.

## 4. Discussion

In this study, we examined the relationship between patient age and resolution of LUTS in patients with PCa with respect to chronological changes in LUTS after LDR-BT, particularly with respect to OABSS. Additionally, we examined the association between OABSS resolution and clinicopathological variables using multivariate analysis. The results in the elderly group were similar to those in the control group with regard to the oncological outcomes of LDR-BT and the rate of resolution of LUTS symptoms, especially the OABSS. Previous studies have reported that scores on the IPSS, OABSS, and IPSS-QOL questionnaires related to urinary symptoms worsened the most at 3 months after LDR-BT; however, the symptoms gradually improved and returned to baseline after 18–36 months [12,13]. In this study, IPSS and OABSS showed the worst worsening 1 month after LDR-BT in the elderly group, whereas similar results were observed 3 months after LDR-BT in the control group. Yamazaki et al. reported that, among patients with PCa who underwent LDR-BT, the proportion of GU toxicity grade ≥2 at 5 years postoperatively was significantly higher in the elderly group (22.1%), compared with 12.7% in the younger group [14]. In this study, regarding LUTS after LDR-BT, the IPSS and OABSS tended to be slightly higher in the elderly group than in the control group, although this difference was not statistically significant between the two groups. The results of this study might be an interesting examination of the importance of carefully monitoring the status of urinary symptoms after LDR-BT in patients with PCa, regardless of the patient’s age, as the results suggest that the change in urinary symptoms after LDR-BT was not related to age.

The treatment modalities for patients with PCa include various treatment options, including open radical prostatectomy (ORP), laparoscopic radical prostatectomy (LRP), robot-assisted radical prostatectomy (RARP), LDR-BT, and EBRT. Several previous studies have reported that posttreatment LUTS have different onset and improvement times depending on the different treatments for PCa [19,20,21,22,23]. Li et al. used the IPSS to investigate ORP, LRP, and LDR-BT with respect to LUTS and reported no significant differences between the three groups during the 8-month postoperative follow-up [19]. Conversely, it was suggested that surgery and radiotherapy might have different types of LUTS, such as stress urinary incontinence (SUI) and urge urinary incontinence (UUI), as well as different onset times, severity, and timing of symptom worsening [20,21,22,23]. Among patients with PCa who underwent RARP, 41% had no preoperative LUTS, whereas SUI, followed by UUI, was most frequently observed postoperatively [20]. The incidence of SUI and UUI decreased gradually at 3, 6, 12, and 24 months postoperatively to 51%, 47%, 41%, and 34%, respectively [20]. Daytime and nighttime frequency and urgency were reported to be persistently observed in 3–5%, 10–15%, and 4–8% after RARP [20]. Regarding voiding symptoms, most patients reported an improvement in the overall IPSS at 12 months after RARP, with a higher IPSS before RARP resulting in higher rates of improvement in voiding symptoms [21]. Although the IPSS improved significantly at 12 months postoperatively, the OABSS did not recover to its preoperative state at 6 or 12 months postoperatively [22]. Age, prostate volume, and preoperative IPSS were associated with improved postoperative LUTS in patients with PCa undergoing RARP, with a trend toward higher LUTS improvement at 12 months postoperatively as patient age increased [23].

In a comparative study of EBRT and LDR-BT with respect to radiation therapy, GI toxicity was reported to be higher with EBRT than with LDT-BT, whereas GU toxicity was found to be higher with LDR-BT than with EBRT, especially in elderly patients [14]. Therefore, careful follow-up may be necessary for postoperative LUTS in elderly patients undergoing LDR-BT. In a report examining the chronological changes in LUTS in patients with PCa who underwent LDR-BT, 72.2–82.1% and 83.3–91.9% of these patients had improved IPSS postoperatively at 12 and 24 months, respectively [24,25]. In a study examining the percentage of patients who recovered their preoperative voiding status after LDR-BT for IPSS, the resolution rates after 24 months were 39.4%, 59.5%, and 83.3% in the mild, moderate, and severe groups, respectively [25]. However, in the Japanese cohort, the resolution rates at 24 months after LDR-BT were 86.6%, 94.8%, and 100%, respectively, which tended to be higher than those reported previously [26]. Additionally, patients with lower baseline IPSS and OABSS required a longer period to resolve urinary symptoms [26]. Furthermore, it has been reported that LUTS, which once improved after LDR-BT, may temporarily worsen again [27,28,29]. When the degree of the second increase in OABSS from the nadir point was evaluated after LDR-BT, the incidence of LUTS relapse was 51.5% and 23.4% for cutoff values of OABSS increases of more than 3 and 6 points, respectively [29]. Although no significantly different variables were detected, there was a trend toward a higher frequency of OABSS relapse in elderly patients undergoing LDR-BT [29]. Furthermore, this secondary exacerbation of urinary symptoms was transient in most patients, although it developed approximately 16–24 months after LDR-BT [27,28]. In contrast, the persistence of LUTS after LDR-BT has been attributed to urinary storage rather than to voiding symptoms [29]. Particularly, it has been suggested that elderly patients with an increase in OABSS of more than 6 points at the time of LUTS relapse may not improve the preoperative voiding status [29]. In this study, a low pre-treatment OABSS was a significant predictor of delayed resolution after LDR-BT. Therefore, the worsening of voiding symptoms after LDR-BT in elderly patients with PCa may be due to preoperative urinary storage symptoms.

Few studies have examined the predictive factors involved in the delayed resolution of IPSS and OABSS after LDR-BT in patients with PCa. Although there have been reports that the total radiation dose to the GU tract affects the delayed resolution of IPSS, others have reported that UD30 was not significantly associated with IPSS [26,30,31]. It has been suggested that the change from baseline with respect to IPSS without concomitant ADT when performing LDR-BT often increases, which may delay the resolution of the IPSS [16,32]. It has been reported that PV is an independent predictor of delayed resolution in OABSS [26]. Therefore, it has been suggested that for patients with a large PV, the administration of ADT may decrease the PV, resulting in a lower incidence of GU toxicity [26]. Considering that concomitant ADT is an independent predictor of reduced GU toxicity, in addition to its likely impact on the early resolution of IPSS and OABSS, ADT might be considered when LDR-BT is performed [32]. In this study, pre-treatment OABSS and OABSS changes from preoperative values were significant predictors of delayed resolution of OABSS after LDR-BT, and the total radiation dose to the prostate, UD 30, PV, and ADT administration were not significantly correlated with the resolution of OABSS. On the other hand, UD30 was lower in the elderly group despite a smaller PV than in the control group. One possible reason for this result may be that the elderly group had more high-risk cases and was, therefore, receiving combined therapy with EBRT. The investigation of factors that predict improvement in urinary status after LDR-BT is a crucial issue to be addressed in the future.

Several limitations of this study are noted. First, the present study was a retrospective, single-institutional, nonrandomized study; thus, the possibility of bias was inherent. Second, the number of enrolled patients was relatively small, and the follow-up period was relatively short. The interpretation of the results obtained in this study should be carefully considered because of the small number of cases in the elderly group. Third, the definition of resolution of the IPSS and OABSS was not clearly determined, and the results may have been ambiguous because the improvement in urinary status was determined based on the judgment of physicians or individual patients. Fourth, we did not collect data on the use of anticholinergic agents or β3-adrenoceptor agonists after LDR-BT; therefore, the effect of these agents on the improvement of voiding symptoms was not examined in this study. Finally, IPSS and OABSS results were obtained from patients, which may have led to uncertainty in the evaluation of LUTS.

## 5. Conclusions

This was a long-term observational study of changes in LUTS after LDR-BT in elderly patients with PCa. To the best of our knowledge, this study is the first to evaluate chronological changes in the IPSS, OABSS, and IPSS-QOL after LDR-BT in elderly patients with PCa. As pre-treatment OABSS and changes in OABSS values before and after LDR-BT were independent factors for predicting a delayed resolution of OABSS, we suggest that the urinary status be carefully monitored when LDR-BT is chosen for elderly patients with PCa who have these conditions. A prospective multicenter clinical trial is required to validate the results of this study, and more elderly patients with PCa should be enrolled.

## Figures and Tables

**Table 1:** Patient characteristics.

	Aged < 75 (*n* = 457)	Aged ≥ 75 (*n* = 27)	*p*-Value *
Age (year, median, IQR)	66.0 (62.0–70.0)	76.0 (75.0–77.0)	<0.001
BMI (median, IQR)	23.5 (21.9–25.3)	23.6 (21.9–25.6)	0.983
Initial PSA (ng/mL, median, IQR)	6.5 (5.0–9.0)	6.6 (5.4–10.2)	0.413
Preoperative prostate volume (mL, median, IQR)	25.0 (18.0–35.1)	21.2 (16.5–29.7)	0.026
Biopsy Gleason grade group (number, %)			0.050
1	195 (42.7)	7 (25.9)	
2	159 (34.8)	8 (29.6)	
3	61 (13.4)	6 (22.2)	
4	29 (6.4)	2 (7.4)	
5	13 (2.8)	4 (14.8)	
Clinical T stage (number, %)			0.688
1	240 (52.5)	12 (44.4)	
2	205 (44.9)	13 (48.1)	
3	12 (2.6)	2 (7.4)	
NCCN risk classification (number, %)			0.045
Low	178 (39.0)	6 (22.2)	
Favorable intermediate	160 (35.0)	7 (25.9)	
Unfavorable intermediate	68 (14.9)	6 (22.2)	
High	48 (10.5)	8 (29.6)	
Very-high	3 (0.7)	0 (0)	
Inserted needles (number, median, IQR)	22 (19–24)	23 (18–24)	0.456
Inserted seeds (number, median, IQR)	64 (52–78)	55 (48–79)	0.485
BED (Gy, median, IQR)	194 (178–210)	198 (180–212)	0.212
D90 (Gy, median, IQR)	157 (127–176)	134 (118–170)	0.174
V100 (%, median, IQR)	97 (95–98)	96 (94–98)	0.685
UD30 (Gy, median, IQR)	192 (160–220)	173 (142–199)	0.005
RV100 (cc, median, IQR)	0.29 (0.07–0.73)	0.18 (0.01–0.64)	0.311
Combined ADT (number, %)	335 (77.7)	20 (74.1)	0.668
Supplementary EBRT (number, %)	183 (40.0)	16 (59.3)	0.051
Preoperative IPSS (median, IQR)	5 (3–9)	5 (3–12)	0.345
Preoperative QOL (median, IQR)	2 (1–3)	2 (1–4)	0.918
Preoperative OABSS (median, IQR)	3 (2–4)	5 (2–5)	0.015
Preoperative Qmax (mL/sec, median, IQR)	17.6 (14.5–21.8)	15.4 (12.9–17.8)	0.019
Change from baseline to maximum in OABSS (median, IQR)	4 (3–6)	4 (2–6)	0.431
Time to maximal OABSS (month, median, IQR)	3 (1–3)	5 (2–6)	0.122
The resolution of OABSS (number, %)	186 (80.2)	11 (84.6)	0.163
Time to the resolution of OABSS (month, median, IQR)	18 (9–48)	12 (9–21)	0.351
Postoperative prostate volume at 3 months after LDR-BT (mL, median, IQR)	13.8 (9.5–20.5)	12.4 (7.2–18.0)	0.175
Postoperative Qmax at 3 months after LDR-BT (mL/sec, median, IQR)	10.1 (7.7–13.7)	8.9 (6.1–12.2)	0.162
Biochemical recurrence (number, %)	9 (2.0)	1 (3.7)	0.312
Follow-up period (month, median, IQR)	76 (42–113)	54 (21–83)	0.014

IQR, interquartile range; BMI, body mass index; PSA, prostate-specific antigen; NCCN, National Comprehensive Cancer Network; BED, biological effective dose; D90, the minimum dose received by 90% of the target volume; V100, percentage of target volume receiving a minimum of 100% of the prescribed dose; UD30, minimal dose received by 30% of the urethra; RV100, rectal volume receiving 100% of the prescribed dose; ADT, androgen deprivation therapy; EBRT, external beam radiotherapy; IPSS, international prostate symptom score; QOL, quality of life due to urinary symptoms; OABSS, overactive bladder symptom score; Qmax, maximal urinary flow rate; LDR-BT, low-dose-rate brachytherapy. * Wilcoxon rank-sum test and Fisher’s exact test.

**Table 2:** Multivariable cox-proportional hazard regression analyses for the resolution of OABSS.

Variable	Univariate		Multivariate	
	HR (95% CI)	*p* Value	HR (95% CI)	*p* Value
Age (continuous)	1.024 (0.998–1.050)	0.071	1.016 (0.991–1.043)	0.220
BMI (continuous)	0.993 (0.937–1.052)	0.815		
Initial PSA (continuous)	0.986 (0.953–1.015)	0.361		
Prostate volume (continuous)	1.000 (0.999–1.001)	0.329	1.001 (1.000–1.002)	0.056
Biopsy Gleason grade group (continuous)	1.015 (0.888–1.151)	0.826		
BED (continuous)	0.995 (0.989–1.001)	0.082		
UD30 (continuous)	0.998 (0.994–1.002)	0.264	1.001 (0.997–1.005)	0.726
Combined ADT (vs. none)	1.304 (0.892–1.907)	0.159		
Supplementary EBRT (vs. none)	0.940 (0.695–1.271)	0.686		
Preoperative IPSS (continuous)	0.998 (0.969–1.027)	0.904		
Preoperative OABSS (continuous)	1.192 (1.115–1.272)	<0.001	1.156 (1.080–1.234)	<0.001
Change from baseline to maximum in OABSS (continuous)	0.767 (0.716–0.820)	<0.001	0.780 (0.728–0.833)	<0.001
Biochemical recurrence (vs. none)	1.119 (0.357–3.509)	0.849		

BMI, body mass index; PSA, prostate-specific antigen; BED, biologically effective dose; UD30, minimal dose received by 30% of the urethra; ADT, androgen deprivation therapy; EBRT, external beam radiotherapy; IPSS, international prostate symptom score; OABSS, overactive bladder symptom score.

## Data Availability

The data presented in this study are available on request from the corresponding author. The data are not publicly available due to privacy and ethical reasons.
